# Exercise programme with telephone follow-up for people with hand osteoarthritis – protocol for a randomised controlled trial

**DOI:** 10.1186/1471-2474-15-82

**Published:** 2014-03-14

**Authors:** Nina Østerås, Kåre Birger Hagen, Margreth Grotle, Anne-Lene Sand-Svartrud, Petter Mowinckel, Eline Aas, Ingvild Kjeken

**Affiliations:** 1 National Advisory Unit on Rehabilitation in Rheumatology, Diakonhjemmet Hospital, Oslo, PO Box 23 Vindern, N-0319 Oslo, Norway; 2 Department of Health Sciences, Institute of Health and Society, Faculty of Medicine, University of Oslo, Oslo, Norway; 3 FORMI (Communication Unit for Musculoskeletal Disorders), Division for Surgery and Neurology, Oslo University Hospital, Ullevål, Olso, Norway; 4 Department of Health Management and Health Economics, Institute of Health and Society, Faculty of Medicine, University of Oslo, Oslo, Norway

**Keywords:** Osteoarthritis, Hand, Exercises, Randomised controlled trial, Study protocol

## Abstract

**Background:**

Hand osteoarthritis (OA) is one of the most prevalent musculoskeletal diseases in an adult population and may have a large influence on an individual’s functioning, health-related quality of life and participation in society. Several studies have demonstrated that exercises may reduce pain and improve functioning in people with knee OA, with a similar effect suggested for hip OA. For hand OA, available research is very limited and shows conflicting results, and high-quality randomised controlled trials are warranted.

This paper outlines the protocol for a randomised controlled trial that aims to determine the effect of an exercise intervention on self-reported hand activity performance in people with hand OA.

**Methods:**

Participants with physician-confirmed hand OA according to the ACR clinical criteria are being recruited from two Norwegian OA cohorts: the population-based “Musculoskeletal pain in Ullensaker Study” (MUST) OA cohort, and the hospital-based Oslo Hand OA cohort. Participants are randomised into an intervention- or control group. The control group receives “usual care”, whereas the intervention group receives a 12-week exercise intervention. The intervention group attends four group sessions and is instructed to perform the exercise program three times a week at home. Adherence will be captured using self-report. During the eight weeks with no group sessions, the intervention group receives a weekly telephone call. The assessments and group sessions are being conducted locally in Ullensaker Municipality and at Diakonhjemmet Hospital, Oslo. Outcomes are collected at baseline, and at 3 and 6 months. The primary outcome measure is self-reported hand activity performance at 3 months post-randomisation, as measured by the Functional Index for Hand Osteoarthritis (FIHOA); and a patient-generated measure of disability, the Patient-Specific Functional Scale (PSFS). Secondary outcome measures are self-reported OA symptoms (e.g. pain, stiffness and fatigue), the Patient Global Assessment of disease activity, measured hand function (e.g. grip strength, thumb web space and hand dexterity) and health-related quality of life. Cost-utility and cost-effectiveness analyses will be conducted.

**Discussion:**

This study will contribute to the knowledge on both the effect and resource use of an exercise programme with telephone follow-up on self-reported hand activity performance among people with hand OA.

**Trial registration:**

The trial is registered at ClinicalTrials.gov with registration number: NCT01245842.

## Background

Hand osteoarthritis (OA) is a common joint disorder that may lead to considerable pain and have a substantial impact on hand function [1]. The consequences of hand OA are of great importance, at the individual level in the form of suffering, reduced function and work ability; and at the societal level in the form of cost for health care and social security. Research on the Framingham Offspring and Community cohorts among women and men aged 28–92 years showed that the prevalence of radiographic hand OA was 51% for women and 48% for men, but that the prevalence of symptomatic hand OA (both radiographic changes and patient-reported symptoms) was 16% and 8% for women and men, respectively [2].

Hand OA may lead to pain both in and around affected joints, and a reduction in joint mobility and grip force, which in turn may result in activity limitations and participation restrictions [1,3]. The clinical manifestations are represented by soft tissue swelling, bony enlargements and bone erosions. These findings occur most frequently in the distal interphalangeal (DIP) and proximal interphalangeal (PIP) joints of the 2nd-5th fingers, as well as in the carpometacarpal (CMC1) joint of the thumb. Previous research indicates that levels of pain and disability are significantly higher among patients with CMC1 involvement, compared to those without CMC1 symptoms [4,5].

At present, no OA disease-modifying interventions are available; therefore, the pharmacological treatment of hand OA is primarily aimed at alleviating symptoms and preventing inactivity and functional loss. International recommendations for OA treatment and standards of care have been developed to improve hand OA management [6,7]. Non-pharmacological approaches are considered to be the core treatment for OA patients [8]; treatment recommendations for individuals with hand OA including functional assessments and instructions in joint protection and work techniques, together with an exercise regimen, thermal modalities and the use of assistive devices, braces or joint supports [6,7].

Although there is a considerable amount of research demonstrating the positive effects of exercise on pain and function in knee OA [9,10], and also partly in hip OA [10,11], research on the effects of exercises in people with hand OA is very limited [12]. Among the few clinical trials that have been done, some have evaluated the effect of exercise in addition to instructions in joint protection and splints [13-15]; whereas four studies have assessed the effect of exercise (including yoga exercises) alone [16-19]. However, in a recent systematic review [12] these studies were rated as having a “high risk of bias” due to methodological limitations such as non-randomisation allocation procedures or small sample sizes. Furthermore, some of the studies reported a positive effect of exercises (and splints) on pain, function or stiffness, while others found no significant effect. Hence, there is conflicting and very limited evidence for the effect of exercises on hand OA, and more and better studies are needed [12,20]. This paper outlines the protocol for an exercise trial with telephone follow-up for people with hand OA.

### Study objective

A randomised controlled trial (RCT) has been designed to investigate the effects of a 12-week exercise programme with telephone follow-up on self-reported hand activity performance among people with hand OA. Our hypothesis is that the intervention group will report better hand activity performance at the 3-month follow-up compared to the control group.

## Methods

### Study development

The study has been designed by a group of researchers with a background as occupational therapists or physiotherapists, and with experience in treating patients with hand OA and in performing clinical trials. Two patient research partners and three primary health-care professionals have also been actively involved in this process.

### Study design

The study has been designed as a pragmatic, assessor-blinded, parallel-group RCT to assess the superiority of a 12-week exercise programme, including telephone follow-up versus usual care at the 3-month follow-up. Measurements are collected at baseline, and at 3- and 6 months post-randomisation. The protocol adheres to the SPIRIT 2013 Statement, which defines standard protocol items for clinical trials [21], and the CONSORT guidelines for non-pharmacological interventions [22,23]. The study is designed to conform to the principles of the Declaration of Helsinki.

### Setting

The study is being conducted in two different Norwegian settings, in the Ullensaker primary health-care services and at Diakonhjemmet Hospital, Oslo. One occupational therapist in Ullensaker and one at Diakonhjemmet Hospital are leading the group exercises at the two locations, and an occupational therapist (ALSS) at Diakonhjemmet Hospital is performing the telephone follow-up. Physiotherapists and occupational therapists in Ullensaker primary health care and at Diakonhjemmet Hospital are conducting the assessments.

### Participants

We aim to recruit 150 persons with hand OA from two different OA cohorts:

1) The Musculoskeletal pain in Ullensaker STudy – Osteoarthritis cohort (MUST OA), a cohort of persons with OA in their hands, hips and/or knees derived from a population-based postal survey sent to all inhabitants between 40 to 79 years of age (n = 12,370) in Ullensaker Municipality in 2010-2011[24]. Approximately 60% of the individuals with self-reported hand, hip or knee OA have attended a comprehensive clinical examination at Diakonhjemmet Hospital.

2) The Oslo Hand OA cohort, which is a cohort of Diakonhjemmet Hospital patients between the ages of 60 and 80 years enrolled between 2000 and 2002 [25].

**Figure 1:**
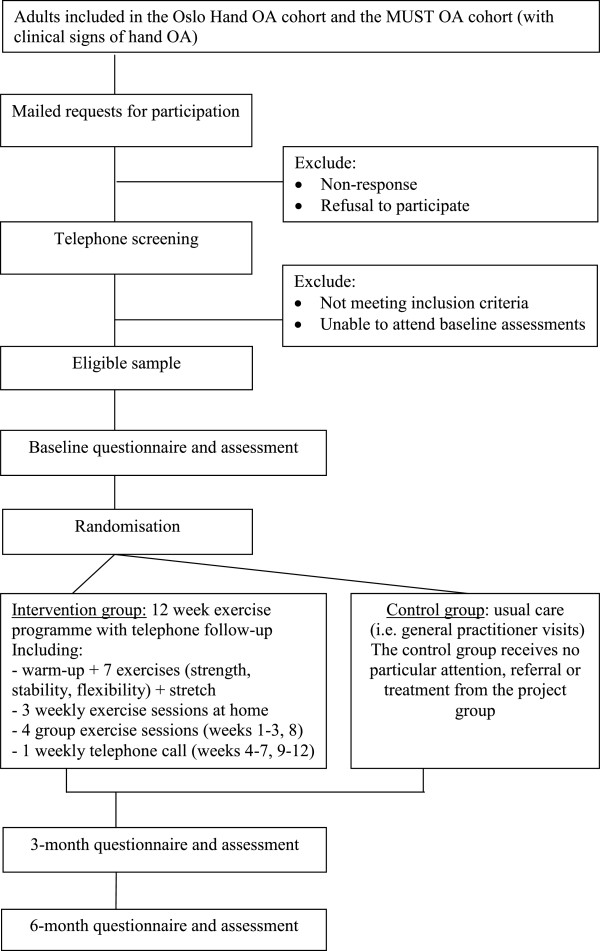
Flow diagram of the study protocol.

Among these, persons with hand OA in accordance with the inclusion and exclusion criteria (Table 1) are being recruited to this RCT, and the recruitment process is outlined in Figure 1. Participant eligibility is checked by the principal investigator (NØ), using the clinical examination data for the MUST OA cohort, and by telephone screening among the Oslo Hand OA cohort. Eligible persons receive written information and a request to participate in the RCT, and baseline measurements are taking place on a regular schedule, with 12–18 participants each time.

**Table 1:** Criteria of inclusion and exclusion

**Inclusion criteria**	**Exclusion criteria**
• Persons with hand OA according to the ACR classification criteria for clinical OA [26]:	• Persons with a cognitive dysfunction;
◦ Pain, aching or stiffness in the hand and 3 of the following:	• Persons who do not understand the Norwegian language;
◦ Hard tissue enlargement involving at least 2 of 10 selected joints	• Persons with inflammatory rheumatic diseases (e.g. rheumatoid arthritis, ankylosing spondylitis) or cancer;
◦ Hard tissue enlargement of at least 2 DIP joints.	• Persons who have recently experienced severe trauma;
◦ Less than 3 swollen MCP joints	• Persons who have recently undergone OA surgery or other major surgery;
◦ Deformity of at least 1 of the 10 selected joints	• Persons who have received steroid injections in their hand joints during the previous two months.
OR uni-/bilateral OA in the CMC1 joint	
• FIHOA* total score ≥5	
• Access to a telephone	

*The functional index for hand osteoarthritis (FIHOA, range 0–30 (0 = best activity performance, 30 = poor activity performance).

### Randomisation and allocation concealment

The randomisation schedule was prepared by the study biostatistician using a computer-generated random numbers table, and randomisation is carried out through the use of random permuted blocks. To help conceal the randomisation, consecutively numbered and sealed opaque envelopes prepared by an independent staff member are used. The envelopes are opened in sequence after the participant has completed all the baseline measurements.

### Blinding

The outcome assessors are blind to group allocation, and are not involved in providing the interventions. The written participant information tells participants that they have an equal chance of being randomised to the intervention versus the control group, but does not provide any details of the actual exercise programme. Allocation is revealed to the three occupational therapists delivering the intervention (group exercises and telephone follow-up), but participants are requested not to disclose details about their group allocation with the outcome assessor. The success of outcome assessor blinding will be evaluated during one group follow-up assessment for the outcome assessors in Ullensaker and one at Diakonhjemmet Hospital by asking the assessors to guess the participants’ group allocation. The statistician who will perform the main statistical analyses will be blinded to group allocation during the analyses.

### Training of research occupational therapists and physiotherapists

The occupational therapists delivering the intervention initially participated in a workshop together with three of the authors (NØ, IK, ALSS) in order to agree on the principles of the exercise programme, and on the information and instructions that should be provided. The outcome assessors participated in a one-hour training session led by the principal investigator (NØ). During this training, the outcome assessors received instructions to screen every completed questionnaire for responder missing while the participant is still present.

### Intervention

Both the control and intervention groups receive “usual care”, which for most people in Norway with hand OA is limited to general practitioner visits (or infrequently, a referral to a consultation with occupational therapists in secondary care). During this study, the control group receives no particular attention, referral or treatment from the project group, and we expect that none, or only a few, will consult a health care professional for their hand OA. Only the intervention group receives the exercise intervention, including the telephone follow-up.

The exercise programme for people with hand OA was developed in 2010 based on the results from a systematic review of the design and effects of splints and exercise programmes, as well as from the American College of Sports Medicine (ACSM) recommendations for developing muscular strength and flexibility in older frail adults [12,27]. The development of the exercise programme followed the new Medical Research Council guidance [28] for developing and evaluating complex interventions, including pilot testing (manuscript under review). Exercises 1–3 aim to increase the muscular strength and stability of the shoulder girdle/upper arm muscles, as this may influence hand activity performance. Exercises 4–5 intend to maintain or increase the flexibility of the MCP, PIP and DIP joints, while exercise 6 aims to strengthen the mm. extensors and abductors pollicis. The purpose is to maintain the thumb web space, increase thumb stability and counteract the strong pull from the adductor pollicis muscle combined with increasing the weakness of the opposing thenar intrinsic musculature, which can be seen in individuals with CMC1 OA, thereby leading to thumb adduction deformity. Exercise 7 intends to increase grip strength. Pipe insulation tubes were chosen instead of balls or other round objects since the tubes allow for squeezing without involving the thumb adductor muscles, which may contribute to a thumb adduction deformity. For the same reason, there is no exercise included to increase the maximum opposition of the thumb (touch the base of the little finger with the thumb tip) or to strengthen the pinch or key grip, as such exercises may accelerate an evolving CMC1-deformity.

The programme starts with a warm-up period consisting of a few minutes of rubbing the hands together and doing arm swings. Exercises 1 – 7 are performed with 10 repetitions for the first two weeks and 15 repetitions for weeks 3 – 12 (Table 2). Thera Bands with different resistance are provided to the participants and tested to identify the Thera Band with an appropriate, individualised resistance. The rubber band is wrapped around the hands or around something else if the hand joints are painful. The participants are instructed to apply a moderate to vigorous intensity in the strengthening exercises and to gradually progress by adjusting the resistance (e.g. by shortening the length of the Thera Bands or changing to a different Thera Band with more resistance).

**Table 2:** Exercise programme for people with hand osteoarthritis

**No.**	Exercise illustration*	**Instructions**
1.		**Shoulder extension:** Sit on an armless chair, knees slightly flexed, and heels on the floor. Start position: hands partly pronated (thumb up), close to the knees. Pull the exercise band back, as the hands follow the thigh to the iliac crest.
2.		**Biceps curl:** Stand with the feet shoulder width apart, arms hanging down. Hands are supinated (thumb laterally). Bend both elbows, pulling the exercise band towards the shoulders.
3.		**Shoulder flexion:** Stand with the feet shoulder width apart, arms hanging down. Hands are pronated (thumb medially). Keep the elbows extended and lift the arms to face level.
4.		**Make an “O-sign”:** Keep the thumb IP and MCP joints slightly flexed throughout. First, open the hand as if grabbing a bottle. Bring the index finger tip to the thumb tip, keeping the MCP, PIP and DIP joints flexed. Open the hand again (“grab the bottle”). Repeat with the 3rd, 4th and 5th fingers.
5.		**Roll into a fist:** First, flex the 2nd to 5th DIPs and PIPs only (keep the MCPs extended). Then flex the MCPs. Hold for 5 seconds. Reverse: extend the MCPs only, then the PIPs and DIPs.
6.		**Thumb abduction/extension:** Put one or more small elastic band(s) around the 1st to 5th proximal phalanges. Rest the loose fist, pronated, on a flat surface. Keep the thumb MCP and IP joints flexed and abduct/extend the thumb. Hold for 5 seconds.
7.		**Grip strength:** Squeeze a pipe insulation tube as hard as possible (isometric hold) for 10 seconds.
8.		**Finger stretch:** Lay the right hand on a flat surface. Use the left hand to apply firm pressure for 30 seconds stretching the 2nd to 5th PIP and DIP joints. Repeat 2 times for each hand.
		If the finger joints are painful: stretch one finger at a time; place the 2nd to 4th finger tips (opposite hand) between the finger joints of the 2nd finger; press for 30 seconds.

*The person on the images has consented for the images to be published.

The intervention group attends one group session per week and does two home sessions per week in weeks 1–3. During weeks 4–7 and 9–12 they do three sessions per week on their own. There is a “booster” group session in week 8 to ensure adherence and to individually adjust the exercises. The group sessions last approximately 45 minutes, and are conducted in the afternoon in the locations of the Ullensaker Primary Health Care and at Diakonhjemmet Hospital. SMS reminders are sent one day in advance, and the intervention group additionally receives a weekly telephone call in the weeks without group sessions (weeks 4–7 and 9–12) for individualised advice that addresses the exercises and encourages programme adherence.

The intervention group keeps the exercise equipment after the intervention period and decides themselves if they want to continue performing the exercise programme. Moreover, no advice is given to the control group at this time, but after the 6-month follow-up, the control group is provided with exercise equipment and instructions in the exercise programme. The Ullensaker cohort will most likely be naïve to hand exercises, while some of the Oslo Hand OA cohort may have received instructions at Diakonhjemmet Hospital Outpatient Clinic in 2007/2008. The Oslo Hand OA cohort control group participants will be asked whether they have received instructions in hand exercises before. At the two follow-ups all participants in the control group will be asked whether they have performed hand exercises in the past 3 months.

#### Telephone follow-up

The complex process of changing one’s own behaviour demands both energy and active involvement, and proceeding as usual often means following the line of least resistance. Thus, individuals need motivating factors and support to continue exercising or to establish the habit of regular exercise [29,30]. Changing health behaviour with education and advice are positive ways of enabling persons to exercise regularly, and there is some evidence which suggests that monthly telephone contact may help improve the clinical status of people with OA [31]. Adherence to the intervention will be targeted during the telephone follow-up using Motivational Interview techniques, a client-centred information and motivation strategy based on cognitive behavioural theory and the trans-theoretical model [32,33]. It is designed to engage ambivalent or resistant clients in the process of health behaviour change, and provides health practitioners with a means of tailoring their interventions to suit the patient’s degree of readiness for change.

### Outcome measures

The outcome measures have been selected based on those recommended for clinical trials in patients with hand OA [34,35], and consist of a combination of patient self-reported outcomes and performance tests. All outcome measures are collected at baseline, 3 months (post-intervention, primary endpoint) and 6 months (Tables 3 and 4). If a participant is unable to attend the assessment on the given date (i.e. due to vacation, sickness, etc.), a new appointment is made. For extraordinary situations (i.e. long-term vacation), the questionnaire is sent by mail/e-mail or the most important outcome measures (marked in Table 3) are collected in a telephone interview.

**Table 3:** Primary and secondary outcomes

**Primary outcome measures:**	**Measurement scale**	Time*
Hand activity performance (The Functional Index for Hand OsteoArthritis, FIHOA [36]) #	0-30	0, 3, 6
Patient-generated disability (Patient-Specific Function Scale, PSFS [37]) #	0-10	0, 3, 6
**Secondary outcome measures:**		
Hand pain #	Numeric rating scale (NRS): 0-10	0, 3, 6
Hand stiffness #	Numeric rating scale (NRS): 0-10	0, 3, 6
Patient Global Assessment of disease activity #	NRS: 0-10	0, 3, 6
Patient Global Assessment of disease activity affecting activities of daily living #	NRS: 0-10	0, 3, 6
Patient Global Assessment of change in disease activity over past three months #	7-point scale	3, 6
Patient Global assessment of change in management of activities of daily living #	5 point scale	3, 6
Grip strength (JAMAR dynamometer)	Kilogramme (mean of three repetitions)	0, 3, 6
Hand dexterity (Moberg Pick-up Test [38])	Seconds (right hand, left hand)	0, 3, 6
Thumb web space (Grip Size instrument)	Cylinder size: 1–12 cm	0, 3, 6
Health-related quality of life (15D [39])	0-1 scale	0, 3, 6

*0 = baseline, 3 = 3 months, 6 = 6 months.

# Items included in the telephone interview when attendance is impossible.

**Table 4:** Tertiary outcome measures

**Tertiary outcome measures**	**Measurement scale**	Time*
Hand pain in which hand	Left/Right/Both	0, 3, 6
Which hand is most painful	Left/Right	0, 3, 6
Hand injury, surgery or injections over past three months	Single question	3, 6
Fatigue	Numeric rating scale (NRS): 0-10	0, 3, 6
Mental distress (General Health Questionnaire, GHQ-20 [40])	Bi-modal fashion (0-0-1-1)	0 months
Self-reported work ability [41]	5-point scale	0, 3, 6
Physical activity (International Physical Activity Questionnaire-Short Form, IPAQ [42])	Mean minutes/week, MET-minutes/week	0, 3, 6
• Vigorous-intensity activity		
• Moderate-intensity activity		
• Walking		
• Sitting		
Exercise self-efficacy [43]	7-point scale	0, 3, 6
Exercise diary (intervention group only)	dd.mm.yy	0-3
• Date	Minutes	
• Exercise duration	NRS: 0–10	
• Pain level post-exercise	Text	
• Comments	Kilogrammes	
• Grip strength at group sessions (weeks 2, 3 and 8)		
Adverse events (intervention group only, obtained from the exercise diary + telephone follow-up log)	Type, duration	0-3
**Direct and indirect costs**		
Self-reported sick leave over past three months	Number of days	0, 3, 6
Absence from non-paid work over past three months	Number of days	0, 3, 6
Self-reported health-care utilisation over past three months: number of visits to general practitioner, medical specialist, physiotherapist, manual therapist, chiropractor, occupational therapist, psychologist, social worker, nurse at general practice/outpatient clinic, “alternative therapy”, hospitalisation	Number of visits	0, 3, 6
Pharmacology use for hand OA over past three months	Self-reported	0, 3, 6
Medical or technical equipment purchased during the past three months	Self-reported	
Costs for attending group sessions (only intervention group): distance travelled, transportation method, transportation costs, work absence, need for accompaniment	Self-reported	0
**Participant characteristic variables**		
Age	Birth year	0
Gender	Female/Male	0
Marital status	Married or cohabiting/Separated or divorced/Widowed/Single	0
Education	Lower secondary school/Higher secondary school/University 1–4 years/University >4 years	0
Employment status	Working full time/working part time/not working/student/working full-time in the home/unemployed or seeking work/age retired/disability pension/sick leave	0
Height	Centimetres	0
Weight	Kilogrammes	0
Dominant hand	Left/Right	0
Year of OA diagnosis	Year	0
Fulfilment of ACR criteria for hand OA	Yes/no	0

*0 = baseline, 3 = 3 months, 6 = 6 months.

### Primary outcome measures

The primary outcome measure is self-reported hand activity performance, as measured by the the Functional Index for Hand OsteoArthritis (FIHOA) [36] and the Patient-Specific Function Scale (PSFS) [37].

The FIHOA consists of 10 items with a four-point Likert scale: “possible without difficulty” (0), “possible with slight difficulty” (1), “possible with important difficulty” (2) and “impossible” (3). In this study, it is used as a patient self-administered instrument, and a total score is calculated, with 0 indicating a good performance and 30 indicating a very poor hand activity performance. The instrument has previously been translated from French to Norwegian using a forward-backward translation that followed recommended procedures [44], and evidence for test-retest reliability, validity and responsiveness has been shown [36,45,46].

The PSFS is a patient-specific instrument commonly used in assessments and evaluations in musculoskeletal pain disorders. The instrument is interview-administered, and the patient is asked to name three to five important activities that they are unable to do or have difficulty doing as a result of their problem (in the present study: their hand OA). The patient then scores the difficulty on an 11-point numeric rating scale (NRS), in which 0 represents “Unable to perform activity” and 10 “Able to perform activity at pre-injury/disease level”. The scores for each activity are used independently and no total score is calculated. In a recent systematic review, the instrument’s measurement properties have been found to be acceptable for several musculoskeletal disorders [47], and it was found to be responsive in patients with musculoskeletal disorders receiving physiotherapy treatment in primary care [48].

### Secondary outcome measures

A number of secondary measures are used (Table 3), including an 11-point NRS to obtain self-reported hand pain, hand stiffness and Patient Global Assessment of disease activity and disease activity affecting the activities of daily living. To help capture potential changes at the 3- and 6-month follow-up, 7- and 5-point versions of the Patient Global Assessment of Change Scales are used to self-report disease activity and the management of activities of daily living, respectively.

Maximal grip strength is measured using a Jamar Dynamometer. The participant sits on an armless chair with the shoulder in a neutral position and the elbow 90° flexed. A maximal squeeze of the dynamometer is performed three times for each hand with 15 second breaks, and the average values for each hand will be calculated.

A functional performance test, the Moberg Pick-up Test, is included to help obtain a quantitative assessment of hand dexterity [38]. The test consists of 12 small objects that have to be picked up while time is recorded using a stop watch. The standard protocol by Ng et al. is applied, and the test is performed once with each hand and the eyes open. The 12 objects are in accordance with a description in the paper by Stamm et al. [38].

To measure the thumb web space in a standardised manner, a Grip Size instrument (12 transparent plexiglass cylinders with a diameter from 1 cm to 12 cm) is used. Participants are asked to grip one cylinder at a time, and the largest size where the assessor can see full contact between the cylinder and the total arch of the participant’s thumb and second digit is recorded.

The 15D instrument of health-related quality of life (15D) is a generic, comprehensive, self-administered instrument that captures 15 dimensions (i.e. mobility, usual activities, discomfort and symptoms, distress) with five response categories in each dimension, thereby making it theoretically possible to further describe the 30 billion health states [39]. A set of utility or preference weights will be used to generate the 15D score (single index number) on a 0–1 scale, which will be used as a utility measure in the cost-utility analysis.

### Tertiary outcome measures

At all three time points, the questionnaire includes questions about bilateral vs. unilateral hand pain and which hand is the most painful. At both the 3- and 6-month follow-ups, the individuals report any arm injury, arm surgery or hand joint injections that have occurred over the past three months. An 11-point NRS is used to capture fatigue, while psychological distress at baseline is measured by the General Health Questionnaire (GHQ-20), a widely used screening instrument for measuring non-psychotic psychiatric illness in a general population [40]. Items are scored as the original GHQ score in a bi-modal fashion (0-0-1-1) [49].

A single question is used to measure self-reported work ability: “To what degree is your ability to perform your ordinary work reduced today?”, with the following response alternatives: hardly reduced at all, not much reduced, moderately reduced, much reduced and very much reduced (score range 0–4) [41].

Physical activity is self-reported using the International Physical Activity Questionnaire-Short Form (IPAQ-SF) [42], which is expressed as weekly energy expenditures determined by the expressed metabolic equivalent task minutes per week (METs min/wk) of different categories (sitting, walking, moderate- and vigorous-intensity physical activity and total physical activity score) and physical activity levels (low, moderate and high).

In order to examine exercise self-efficacy, a self-administered questionnaire with 12 statements is used to evaluate how certain the participants are that they are capable of sticking to the exercise programme, even under unfavorable circumstances [43]. The instrument has a 7-point response scale (range 1–7, higher scores reflect higher exercise self-efficacy), but only the first-, middle- and last response options are worded: “Not certain at all”, “Maybe” and “Very certain”, and a mean score is calculated.

The intervention groups are asked to keep an exercise diary that includes a registration of the date and the duration of each exercise session. The individuals report their pain level post-exercise and write comments if any. Maximal grip strength is measured at the group sessions by one repetition for each hand after warm-up, but before performing the exercise programme. Based on the diary notes, the total number of exercise sessions performed will be calculated, and a high compliance will be defined as attendance at more than 75% (three of four) of scheduled group exercise sessions and 60% (22 of 36) of prescribed home exercise sessions in the intervention group, as described in a previous hip OA exercise trial protocol [50].

### Adverse effects or events

Adverse events related to exercises for people with hand OA are not consistently studied, but the risk is considered to be low if the suitability of the exercise for the individual is appropriately assessed by a trained health professional [8,51]. Should they occur, adverse events related to the exercise intervention are documented by the type and duration, and information on this is collected and registered during telephone counseling and from the exercise diary.

#### Direct and indirect costs

Based on a validated cost diary and a previous study [52,53], a questionnaire was developed to collect information on resource use. Number of sick leave days and absence from non-paid work is reported over the previous three months. Furthermore, health-care utilisation for the previous three months is self-reported as number of visits to relevant health-care providers. They also report any medication taken for hand OA, as well as medical or technical equipment purchased during the past three months. The intervention group fills in an extra questionnaire targeting costs for attending the group sessions (i.e. travel distance, transportation method, public transportation cost if applicable, work absence or travel escort).

### Statistical analyses

#### Data analysis

Demographic and clinical characteristics as well as other baseline data are being collected and will be presented to assess the baseline comparability of the two groups. These variables will also be compared for those participants who withdraw from the study and those who remain. Parametric and non-parametric statistical analysis models will be used depending on the distribution of the variables.

The intention-to-treat principle (ITT) will be followed in the primary analyses of data, and will include all participants, also those who have missing data and those who are not fully compliant with the protocol. Descriptive statistics will be presented for each group as the mean change (standard deviation, 95% confidence intervals) in the outcomes from baseline to each time point. Differences in mean change from baseline to each time point will be compared between groups, using linear mixed models or generalised linear regression modeling, adjusting for baseline levels of the outcome measure. Model assumptions will be checked by standard diagnostic plots, and improvements in the intervention and control group based on the perceived ratings of change will be compared using logistic regression, and presented as odds ratios with 95% confidence intervals. Additionally, the number of “responders” in the two groups will be compared using the OMERACT-OARSI responder criteria [54]. A participant will be classified as a responder if one of the following is fulfilled:

1) High improvement:

•≥50% improvement + absolute change of ≥2 in self-reported hand pain (NRS, 0–10), OR

•≥50% improvement + absolute change of ≥6 in self-reported hand activity performance (FIHOA, 0–30);

OR

2) Improvement in at least 2 of the 3 following:

•≥20% improvement + absolute change ≥1 in self-reported hand pain

•≥20% improvement + absolute change ≥1 in Patient Global Assessment of disease activity (NRS, 0–10)

•≥20% improvement + absolute change ≥6 in self-reported hand activity performance (FIHOA)

An exploratory sub-group analysis will be completed to examine the effectiveness of the exercise programme for those participants attending all group sessions and performing all home sessions. This analysis will only be completed if there are sufficient participants attending all four treatment sessions and performing all home sessions. Treatment concordance will also be evaluated descriptively by self-reported exercise frequency and duration at the 3- and 6-month follow-up.

Exact analyses utilising the binominal distribution will be used to assess the success of outcome assessor blinding (with 95% confidence intervals); no statistical adjustment will be made for multiple testing, and all tests will be two-sided and carried out at the 5% level of significance. Any changes to the study design or analysis plan will be documented with full justification.

### Health economics

Based on the findings in this study two economic evaluations will be conducted, applying both health system and societal perspective. Costs in the health-care sector comprise intervention costs and costs related to treatment and follow-up, while societal costs include production loss, as well as costs for the individuals and the family. The primary economic evaluation will be cost-utility analysis (CUA) of the cost per extra quality adjusted life years (QALYs), which will be calculated using the 15D scores at 3 and 6 months. The secondary evaluation will be a cost-effectiveness analysis (CEA) based on the disease-specific measure FIHOA. Incremental cost per QALY (cost per FIHOA) will be calculated as the ratio of the difference between groups in mean cost to the difference in mean QALYs (FIHOAs). By means of bootstrapping, cost-effectiveness acceptability curves (CEACs) were used to consider the uncertainty surrounding the cost-effectiveness of the exercise programme by plotting the probability that exercise programme is cost-effective according to a range of willingness-to-pay thresholds.

### Sample size

The primary outcome measure FIHOA sum score (range: 0–30 points) was used to estimate sample size. Using a minimal clinically important change (MIC) of three points (10%) (sd 6.2), with a significance level of 0.05 (2 tailed) and a power of 80%, we estimated that 68 persons will be needed in each group. To allow for a 10% drop-out rate, 75 persons will be included in each group.

### Time schedule

3rd quarter 2010 – 4th quarter 2012: Recruitment of study participants;

1st quarter 2011 – 1st quarter 2013: Baseline measurements, exercise intervention and telephone follow-up;

4th quarter 2010 – 2nd quarter 2013: The follow-up measurements;

2nd quarter 2013 – 4th quarter 2013: Data analysis, writing and submitting articles.

### Ethics

The study is conducted according to good clinical practice, and is in compliance with the Declaration of Helsinki; and the study was approved by the Regional Committee for Medical and Health Research Ethics (Ref. no: 2010/727a) and the Data Inspectorate. The study participants receive written and oral information about the study, and written consents are collected prior to the baseline data collection.

## Discussion

The prevalence of hand OA increases with age, and is growing due to the aging of the population. Since there are no disease-modifying interventions available and pharmacological treatment of hand OA is primarily limited to symptom relief, the effectiveness of non-pharmacological treatment modalities should be further explored and documented. Although exercise is recommended as a core treatment for people with OA, to date, research on the effect of exercise has mainly been performed in people with knee OA, and also hip OA to a lesser degree. However, available research assessing the effect of exercises in people with hand OA is very limited and shows conflicting findings. Lack of gain in muscle strength in exercise programmes designed to improve grip strength has been previously reported [17,19], whereas a programme with flexibility exercises resulted in increased grip strength [15].

Hence high-quality randomised controlled exercise trials for this patient group are warranted. This paper outlines a protocol for a pragmatic RCT, including an exercise intervention with telephone follow-up for people with hand OA.

Among the strengths of this trial is the implementation of an exercise programme developed following the new Medical Research Council guidance for developing and evaluating complex interventions [28,42]. The development followed a systematic approach, using the current evidence, expert opinions, and involving patient research partners in designing the study and piloting the intervention. The exercise programme is relatively short, and the equipment required is cheap and easily available for health care professionals and individuals with hand OA. Further research is now needed to investigate the effectiveness of this exercise programme in people with hand OA.

The intervention effects will be assessed with a combination of validated-, self-reported patient outcome measures and performance-based observation tests. The trial measure outcomes for clinical trials recommended by Osteoarthritis Research Society International (OARSI) and Outcome Measures in Rheumatological Clinical Trials (OMERACT) are included in the study. Additionally, we will record and evaluate the success rate of the assessor blinding strategy, and perform cost-utility- and cost-effectiveness analyses to assess the costs and effects of the exercise intervention compared to usual care.

Some attrition is anticipated despite the fact that we have planned procedures to minimize loss due to follow-up and participant withdrawal, and to maximize adherence. This may represent a limitation insofar as the planned trial does not include a placebo or attention control intervention for the control group. However, no optimal solution for the content or delivery of a “sham exercise intervention” was identified and an educational intervention for the control group might have resulted in limited contrasts between the two groups. Hence, this study is designed as a pragmatic trial comparing the active intervention with the usual care.

In conclusion, this study will contribute to the knowledge of the effect of exercises with telephone follow-up on self-reported hand activity performance in people with hand OA, in addition to the cost-utility and cost-effectiveness of the exercise intervention.

## Abbreviations

ACR: American college of rheumatology; CMC: Carpometacarpal; CEA: Cost-effectiveness analysis; CEAC: Incremental cost-effectiveness acceptability curves; CUA: Cost-utility analysis; DIP: Distal interphalangeal; FIHOA: Functional index for hand osteoarthritis; GHQ: The general health questionnaire; ICER: Incremental cost-effectiveness ratio; IPAQ-SF: International physical activity questionnaire –short form; ITT: The intention-to-treat principle; MCP: Metacarpophalangeal; MIC: Minimal clinically important change; MET: Metabolic equivalent task; MUST: Musculoskeletal pain in ullensaker STudy; NICE: National institute for clinical excellence; NRS: Numeric rating scale; OA: Osteoarthritis; OARSI: Osteoarthritis research society international; OA-QI: OsteoArthritis quality indicator questionnaire; OMERACT: Outcome measures in rheumatological clinical trials; PIP: Proximal interphalangeal; PSFS: Patient-specific functional scale; QALY: Quality adjusted life years; RCT: Randomised controlled trial; 15D: The 15D instrument of health-related quality of life.

## Competing interests

The authors declare that they have no competing interests.

## Authors’ contributions

IK and MG conceived the project idea and designed the study together with NØ, KBH and ALSS. NØ administer the trial and coordinate the assessments. IK and NØ have trained the therapists involved in the assessments and provision of the intervention. NØ recruit and screen the participants and ALSS perform the telephone follow-up. NØ drafted this manuscript, and EA drafted the health economic section of the manuscript. All the authors provided feedback on the drafts, and have read and approved the final manuscript.

## Pre-publication history

The pre-publication history for this paper can be accessed here:

http://www.biomedcentral.com/1471-2474/15/82/prepub
